# Estimating the Instantaneous Frequency of Linear and Nonlinear Frequency Modulated Radar Signals—A Comparative Study

**DOI:** 10.3390/s21082840

**Published:** 2021-04-17

**Authors:** Hubert Milczarek, Czesław Leśnik, Igor Djurović, Adam Kawalec

**Affiliations:** 1 Faculty of Electronics, Military University of Technology, ul. Gen. Sylwestra Kaliskiego 2, 00-908 Warsaw, Poland; hubert.milczarek@wat.edu.pl; 2 Electrical Engineering Department, University of Montenegro, Cetinjski Put 2, 81 000 Podgorica, Montenegro; igordj@ucg.ac.me; 3 Faculty of Mechatronics, Armament and Aerospace, Military University of Technology, ul. Gen. Sylwestra Kaliskiego 2, 00-908 Warsaw, Poland; adam.kawalec@wat.edu.pl

**Keywords:** electronic support measures, electronic intelligence, radar signal, modulation recognition, intrapulse analysis, instantaneous frequency, linear frequency modulation, nonlinear frequency modulation, multipath

## Abstract

Automatic modulation recognition plays a vital role in electronic warfare. Modern electronic intelligence and electronic support measures systems are able to automatically distinguish the modulation type of an intercepted radar signal by means of real-time intra-pulse analysis. This extra information can facilitate deinterleaving process as well as be utilized in early warning systems or give better insight into the performance of hostile radars. Existing modulation recognition algorithms usually extract signal features from one of the rudimentary waveform characteristics, namely instantaneous frequency (IF). Currently, there are a small number of studies concerning IF estimation methods, specifically for radar signals, whereas estimator accuracy may adversely affect the performance of the whole classification process. In this paper, five popular methods of evaluating the IF–law of frequency modulated radar signals are compared. The considered algorithms incorporate the two most prevalent estimation techniques, i.e., phase finite differences and time-frequency representations. The novel approach based on the generalized quasi-maximum likelihood (QML) method is also proposed. The results of simulation experiments show that the proposed QML estimator is significantly more accurate than the other considered techniques. Furthermore, for the first time in the publicly available literature, multipath influence on IF estimates has been investigated.

## 1. Introduction

Recent years have seen dynamic development in radar instruments, including advancement in multiple-input multiple-output (MIMO) techniques, the pursuit to put quantum sensors into practice and the continual exploitation of even more ingenious modulation types. The last fact could not be overlooked from the context of electronic warfare (EW). This activity is aimed at gathering information about adversary radars, which is usually accomplished by highly sensitive interception receivers, specialized in collecting signals emitted by far, non-cooperative emitters. During peacetime, intercepted radar signals may be analyzed to find out the purpose and capabilities of the specific device. By contrast, during a conflict, the detection, identification and geolocation of enemy radar becomes far more important. The acquired intelligence can then be utilized to target or penetrate hostile air defense. Another relevant function of interception systems is supplying emitter data to databases used by other EW systems, such as radar warning receivers (RWRs).

Traditionally, electronic interception assets are divided into two distinct categories: electronic support measures (ESMs) and electronic intelligence (ELINT), where the former are intended for deployment on a tactical level in order to provide situational awareness, and role of the latter is to conduct long-term analysis and provide national level strategic databases. In recent years, the line between both systems has become blurred, since modern ESM systems are now capable of performing tasks distinctive for ELINT, including the recording of intercepted signals [1] (p. 4). For this reason, here, both kinds of the systems are referred jointly as ESMs.

Commonly, signal emitted by surveillance radar has the form of pulse train. Thus, an ESM system performs both inter-pulse and intra-pulse analysis in order to determine the so-called pulse descriptor word (PDW), while a set of PDWs forms a signal signature unique to a certain type of radar. Nowadays a PDW is supplemented with information on modulation observed in the intercepted signal, i.e., modulation on pulse (MOP). This extra characteristic eases the attribution of a PDW to a given emitter (a task which can become quite challenging in a dense electromagnetic environment) but also aids pulse deinterleaving algorithms or may be exploited during the assessment of the application and performance of the unknown radar. Today’s ESM systems are capable of determining MOP in real-time, with no human involvement needed, by means of automatic modulation recognition/classification algorithms (AMR/AMCs).

AMC techniques can be generally divided into two distinct categories: one is statistical hypothesis testing and the another is pattern recognition via extracted signal features [2]. Examples of the former approach can be found in papers [3,4]. However, the vast majority of existing algorithms employed pattern recognition methods. This implies the selection of features distinctive to a certain modulation, the devising of a mechanism able to extract the features from the signal and consecutively supplying extracted features to a decision-making algorithm (classifier), which assigns an unknown signal to one of the known modulations. The most common classifiers in radar AMC algorithms are hierarchical decision trees and artificial neural networks, whereas there are a lot of possible signal features, including those derived from instantaneous signal properties [4,5,6], higher-order statistics [6,7,8], time-frequency distributions (TFDs) [8,9,10] or power spectral density [11,12]. Over the recent years, one can notice an increasing number of deep learning AMC algorithms, especially those using time-frequency image features combined with a convolutional neural network classifier [13,14,15].

Much effort has been devoted yet to recognize miscellaneous radar signals. Numerous studies have concerned widespread waveforms, such as linear frequency modulation (LFM) or binary phase shift keying (BPSK). Nonetheless, in the case of another prominent waveform which is nonlinear frequency modulation (NLFM), the number of devised methods is very restricted. Examples of such AMC algorithms, which distinguish NLFM as a separate class, may be found in [16,17]. However, both papers examine only the single NLFM waveform. Besides two mentioned works, there are a couple of distinct reports regarding NLFM recognition, yet they are questionable because they assume the NLFM model, which substantially differs from one used in real-life radars.

An intuitively appealing feature of the NLFM signal is its instantaneous frequency (IF), which should clearly separate this waveform from the others. Methods and algorithms for estimating the IF of noise-corrupted signals is a substantial field of knowledge, which has been developed for over three decades [18,19]. Meanwhile, this issue is not addressed in the context of AMC algorithms with a few exceptions in [4,20,21]. Consequently, different authors apply arbitrarily chosen IF estimation methods in the feature-extraction stage, without examining their accuracy in prior. It is worth stressing that effective features can offset the mediocre performance of the classifier [22] (p. 11) and fairly improve overall classification results. The purpose of this study is to investigate different methods practiced for IF estimation with regard to linear and nonlinear frequency modulated radar signals.

The contributions of this paper are as follows:

1. It is the first extensive comparison of five different instantaneous frequency estimation methods which are specific to radar signals recognition, which also accounts for various frequency modulation laws.
2. We introduce an alternative IF estimation algorithm based on the generalized quasi-maximum likelihood (QML) method with a novel signal model.
3. Multipath propagation effects inherent to ESM analysis has been discussed and their influence on respective estimation methods is examined.

The paper is organized as follows. Section 2 presents the recognition system model as well as the assumed signal model. A multipath phenomenon description is introduced in Section 3. Section 4 gives the considered IF estimation methods. Section 5 shows the simulation experiments’ results. Finally, Section 6 discusses the paper and presents the conclusions.

## 2. System and Waveforms

**Figure 1:**
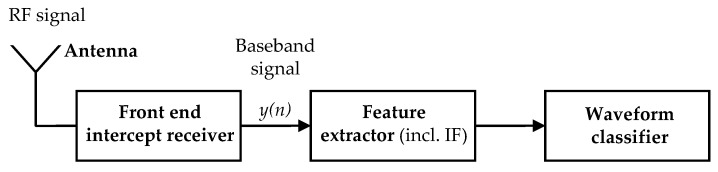
Block diagram of the typical radar waveform recognition system.

A typical automatic modulation recognition algorithm in an ESM system may be divided into the feature extraction stage and classifier stage, as shown in Figure 1. Here, we assumed a common situation, i.e., that one of the analyzed signal features is the IF. First, the signal was detected and then carrier frequency was estimated and removed. Hence, a complex envelope was determined. The time of arrival and pulse duration were assumed to be known. The AMC algorithm itself is not considered in this paper.

A feature extractor is supplied with a discrete-time radar pulse corrupted with noise y(n) and sampled with rate $f_{s}$. The signal model is then given by

(1) $$y(n)=x(n)+\varepsilon (n)=a(n)e^{j\phi (n)}+\varepsilon (n),$$

where x(n) denotes the complex radar signal, n is the sample index, $T_{s}$ stands for sampling interval $x(n)=x(nT_{s})$, ε(n) represents complex additive white Gaussian noise (AWGN) with variance $\sigma_{\varepsilon }^{2}$ and a(n) with ϕ(n) are instantaneous amplitude and instantaneous phase, respectively.

In reality, the pulse amplitude is not constant during the time, as both the rising and falling edge have some finite time. This phenomenon is usually caused by the transmitter, but the envelope can be also tapered deliberately in order to limit out-of-band emissions or suppress the sidelobes in matched filter. In the literature, the instantaneous amplitude of a pulse is frequently modeled by weighting functions such as the Tukey or raised cosine window. Here, similarly to [23], we adopted the Tukey window with the shape parameter $\alpha_{T}=0.05$.

This paper is focused on nonlinear frequency modulated radar signals. Along with LFM, it is one of the first conceived radar waveforms. NLFM’s advantage over LFM comes from its naturally low sidelobes level, attainable without a need of mismatched processing, which is not the case for LFM. Thanks to this, the entire transmitter energy can be embraced. This is reflected in growing interest in NLFM, especially for synthetic aperture radars [24] or long-range surveillance radars [25]. The last decade brought rapid advancement in NLFM synthesis methods, with algorithms based on Bézier curves [26] or genetic optimization techniques [27]. The vast majority of already devised methods have one thing in common, namely that yielded IF functions are monotonic, symmetric with respect to time point corresponding to center of the pulse and the chirp rate is abruptly increasing towards both edges of the pulse. Different, i.e., nonsymmetrical IF generally would result in a deteriorated sidelobes level and Doppler tolerance [26] so it is hard to find such forms of NLFM in real-life applications. If we designate the constant-time variable as t, pulse duration as T, frequency deviation as ΔF, and IF as f(t) and further assume that pulse is defined in time period [−T/2,  T/2], it may be concluded that the IF of the NLFM signal meets the following assumptions:

(2) $$\begin{array}{c} f(-T/2)\;=\;-\Delta F/2,\\ f(t)\;=\;-f(-t),\\ \left|df/dt\right|\;>0. \end{array}$$

The following NLFM signal synthesis methods were used in the remainder of this study:

1. Based on the stationary phase principle (SPP). It is a primary design approach, originally devised by Fowle in the 1960s [28]. The IF is determined using its approximative relationship with inversed group delay, which in turn is obtained from the assumed prototype of energy spectral density. An interested reader can find a concise explanation of the method in [29] (pp. 87–89). Here, during the synthesis process, we picked Blackman–Harris due to its ability to achieve low sidelobes while preserving adequate range resolution [30].
2. A design algorithm which describes the instantaneous phase of the NLFM waveform as a combination of the catenary and kappa curve [31]. In experiments, we used the optimization parameter α=6 as defined in [31] and assumed that the maximum Doppler shift $f_{D}=6\;\mathrm{kHz}$.
3. The NLFM waveform originally proposed for active sonar [32]. For our study, we skipped additional amplitude tapering and applied parameters of frequency modulation function α=0.52 and γ=1.47 as defined in [32].

Furthermore, the LFM was also employed in research for comparison.

**Figure 2:**
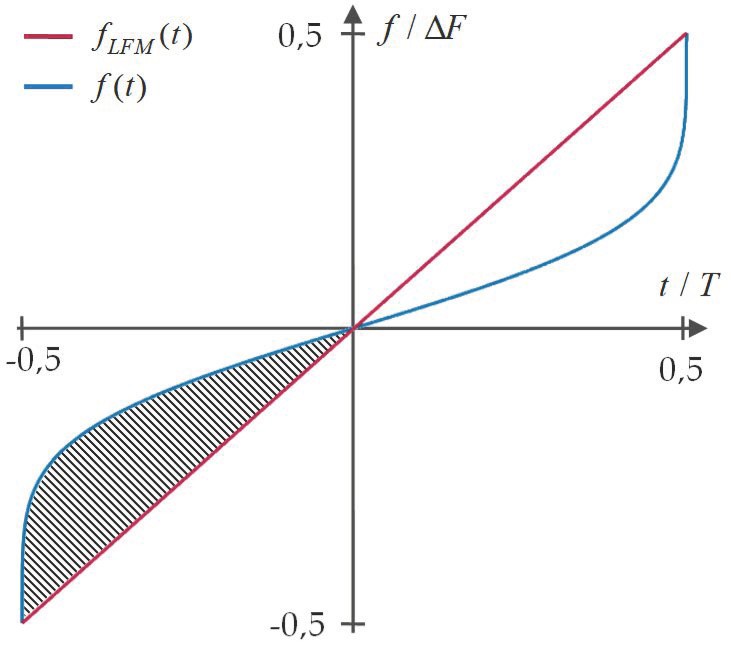
Illustration of nonlinearity factor (in above case η=0.57).

One can note that frequency variations in the NLFM waveform can have different sizes, depending on specific design requirements. For this reason, it is helpful to introduce the notion of nonlinearity factor, given by

(3) $$\eta =8\;\int_{-T/2}^{0}\left[\frac{f(t)-f_{LFM}(t)}{\Delta F}\right]dt,$$

where $f_{LFM}(t)$ denotes the IF of the LFM signal with the same pulse duration, frequency deviation and modulation direction (here we assumed that both signals are upchirp) as the analyzed NLFM signal. Multiplication by eight is used to fit the coefficient values to the interval [0, 1], where 0 represent signals with purely linear frequency modulation and 1 describes signals which exhibit sharp frequency variations on the pulse borders (the waveform proposed by Price [29] (p. 92) may be used as an example of such). The nonlinearity factor can be simply seen as a value proportional to the area between the normalized IF of the NLFM and LFM signals, as illustrated in Figure 2.

The comparison of different NLFM waveforms in the function of factors such as a time–bandwidth product (TBP) is further complicated by the fact that for the NLFM signal, the frequency deviation is not equal to the effective signal bandwidth B (generally, deviation is larger). For this reason, in experiments we assumed that the instantaneous frequency took the values from the interval f(t)∈[−ΔF/2, ΔF/2], which are adjusted in order to fit the required bandwidth. Meanwhile, the bandwidth is interpreted here as the frequency span which contains the majority of the signal energy. To facilitate this, the in-bound energy ratio parameter (IBER) may be used, introduced in paper [33] and defined as

(4) $$\mathrm{IBER}=\frac{\int_{-B/2}^{B/2}\left|X(f)\right|df}{\int_{-\infty }^{\infty }\left|X(f)\right|df},$$

where |X(f)| represents the amplitude spectrum of a signal. IBER = 0.9 is used in simulation experiments.

Finally, all radar waveforms employed in the study are summarized in Table 1.

## 3. Multipath Phenomenon

**Figure 3:**
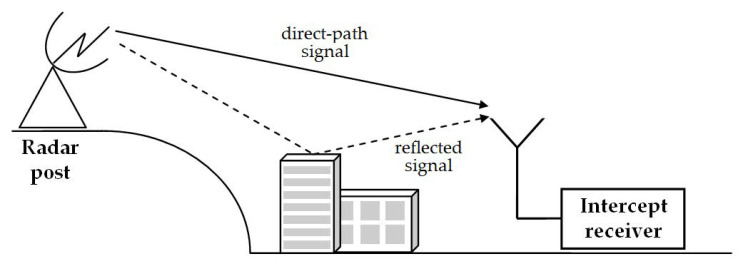
Schematic explanation of multipath phenomenon with a single reflection.

Commencing the analysis of intercepted radar signals, one often has to deal with the multipath phenomenon. It comes up when the interception system receives unwanted signals reflected from the ground, trees, buildings, etc., in addition to the desired direct-path signal. Reflected signals make a detour, so they are delayed and will consequently have a different phase than the direct-path signal. The resultant signal perceived by the receiver is the effect of constructive and destructive interferences of the direct-path and reflected signals, the interferences which particularly distort the envelope. The instantaneous amplitude may increase or droop, and in extreme cases even wane. The multipath phenomenon is illustrated in Figure 3.

Side effects which come from the multipath were thoroughly investigated in the context of global navigation satellite systems (GNSS) and wireless communications. Nonetheless, multipath is averse to the ESM systems as well, while its most recognizable outcome is the deterioration of direction-finding algorithms. Until recently, only a few studies have taken up this subject, such as [34] (p. 266). Meanwhile, significant contribution was made by Robertson in her book [1]. Here, we adopt a simplified description of the phenomenon, yet sufficient to evaluate the considered algorithms. Inspired by the profound DMCM model in [35], if we assume that each signal cluster consist of one propagation path, both the radar and ESM platform are stationary and no local scattering is present, the pulse affected by the multipath $y_{M}(t)$ can be represented as a vector sum of the direct-path pulse $y_{D}(t)$ and its Z reflected copies $y_{R}(t)$:

(5) $$y_{M}(t)=y_{D}(t)+\sum_{z=1}^{Z}y_{R_{z}}(t)=a(t)\exp\left[j\phi (t)\right]+\sum_{z=1}^{Z}\lambda_{z}\zeta_{z}a(t-\Delta t_{z})\exp\left\{j\left[\phi (t-\Delta t_{z})+\Delta \phi_{z}\right]\right\},$$

where λ<1 denotes the reflection coefficient which is related to the attenuation level at the reflection surface and depends on the grazing angle, the polarization of the reflected signal and the admittance of the reflecting medium [1]. Further, Δt is the time lag between the direct-path signal and reflected signal (for the intra-pulse distortions to occur Δt<T). Clearly, reflected rays will be delayed with respect to the direct-path signal, making a detour of ΔR=c Δt, where c stands for speed of light in vacuum. Next, ζ denotes the amplitude attenuation factor accounting for path loss, whereas power attenuation is given by $P_{dB}=20\log\zeta$ [36]. Following power delay profiles remarked in [35], here we assume that power decreases linearly with respect to distance [36] and $P_{dB}=-0.03\Delta R$. Next, Δϕ represents the phase difference between the direct and reflected signal, and z is a number of the respective reflected pulse.

The multipath phenomenon is commonly described by statistical means. In simulation studies, we assumed that the phase has a uniform distribution as propagation delays are usually much greater than the carrier signal period, so all phase values are equally likely. Next, for a given wavelength, the reflection coefficient strongly depends on the reflection geometry and scattering medium [1]. As we do not model any specific reflectors nor spatial scenario, we adopt that λ is governed by the uniform distribution. Lastly, we assume that time delays Δt follow Gamma distribution, as found in [36]. This observation relates to GNSS, albeit in the public domain there are not adequate studies which specifically concern ESM.

**Figure 4:**
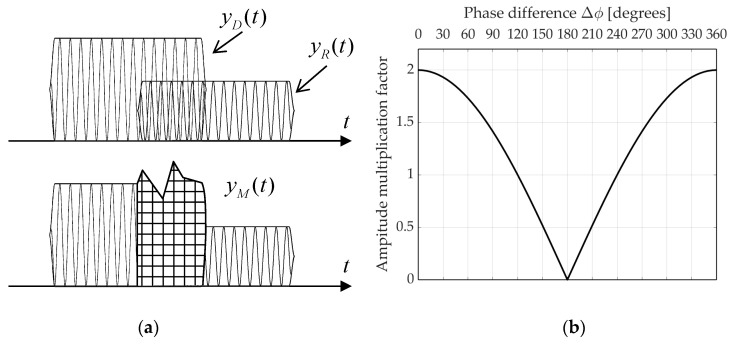
(**a**) Diagram showing how the multipath affects the pulse envelope; (**b**) amplitude multiplication factor for the combination of two equal amplitude signals [1]. The graph relates to the result of two constant-frequency pulses combined. In the case of chirped pulses, a multipath will cause abrupt variations in the amplitude profile during the time.

A diagram of the multipath phenomenon for a single reflection is depicted in Figure 4a. It should be noted that the phase difference between consecutive signals determines whether interferences will take a destructive or constructive form, as can be inferred from Figure 4b.

## 4. Considered IF Estimation Methods

In radar AMC algorithms, there are two widely used IF estimation techniques: based on phase finite differences and time-frequency distributions. Here, we scrutinize both approaches, considering five distinct estimation methods. Three of them are variants of phase differentiation techniques (Section 4.1), two employ TFDs (Section 4.2 and Section 4.3) and the remaining is based on the generalized QML method (Section 4.4).

### 4.1. Phase Finite Differences

The IF of a signal is a derivative of its instantaneous phase ϕ(t) with respect to time, i.e.,

(6) $$f(t)=\frac{1}{2\pi }\frac{d\phi (t)}{dt},$$

whereby for a discrete-time analytic, a signal’s instantaneous phase can be easily determined as an inverse tangent of the ratio of quadrature and the in-phase component. Phase function should be unwrapped in prior, i.e., its absolute jumps towards π should be replaced by their ±2π complements.

After the discrete, unwrapped instantaneous phase of the intercepted signal ϕ(n) is obtained, the IF estimate $\hat{f}(n)$ can be easily calculated using a popular numerical method for solving differential equations, namely by a finite difference of a form

(7) $$\hat{f}(n)=\frac{1}{2\pi }\left[\frac{\phi (n)-\phi (n-1)}{T_{s}}\right].$$

The aforementioned method is termed as backward finite difference (BFD). It was referred to both in the ESM literature [34] (p. 270) and afterwards exploited in numerous AMC algorithms [7,8,16,37] for feature extraction of a signal.

Similarly, after applying a minor amendment in the above formula, a central finite difference (CFD) estimator is yielded. It is unbiased, does not introduce group delay for LFM signals [18] and can be noted as

(8) $$\hat{f}(n)=\frac{1}{2\pi }\left[\frac{\phi (n+1)-\phi (n-1)}{2T_{s}}\right].$$

Both mentioned IF estimators exhibit high variance while dealing with distinct noise. There are a couple of solutions to limit the variance. One of them is to average or “smooth” successive phase differences in the moving window. The estimator proposed by Kay (hereinafter referred to as KAY) may serve here as an example [38]

(9) $$\hat{f}(n)=\frac{1}{2\pi }\sum_{i=0}^{N-2}w(i)\left[\frac{\phi (n+1+i)-\phi (n+i)}{T_{s}}\right],$$

with an averaging window of a length N−1, formed by

(10) $$w(i)=\frac{1.5N}{N^{2}-1}\left\{1-{\left[\frac{i-\left(\left(N/2\right)-1\right)}{N/2}\right]}^{2}\right\}.$$

Due to windowing, the variance of the estimate is reduced by approximately N/6 [18]. In simulation experiments, we adopted N=16, which assures a substantial error reduction. The application of a considerably longer window is inadvisable since the estimate will be degraded if rapid frequency variations are present within the window [18].

### 4.2. Data Driven Pseudo-Wigner Distribution

Another prevalent method of IF estimation is to extract the ridge from the time-frequency image of a signal [18,19]. Authors of the AMC algorithms also draw from this approach, using different forms of Wigner–Ville distribution (WVD), as can be seen in [11,39]. Next, the IF estimator in consideration belongs to this category. In the first step, a time-frequency image is obtained through pseudo-Wigner distribution (PWD) with an adaptive window length. The discrete PWD is described by the formula [40]

(11) $$W_{PW}(n,k)=\sum_{m=-N}^{N-1}w(m)y(n+m)y^{*}(n-m)e^{-j4\pi mk/2N},$$

where N is a positive integer, k is the frequency index and w(m) denotes the real-valued window of length 2N. The idea behind the algorithm is to select length of the PWD window independently for each time instant, in order to reach best bias–variance tradeoff. An interested reader may find its details in [40]. In simulations, we used a rectangular window with lengths ranging from 10 to 128 samples.

Wigner distribution has the best energy concentration among quadratic TFDs [41] (p. 585). PWD also exhibits good concentration and therefore the algorithm assumes that for a given time instant, the IF estimate can be simply obtained from the peak of the time-frequency distribution, i.e.,

(12) $$\hat{f}(n)=\arg\underset{k\in Q_{k}}{\max}W_{PW}(n,k),$$

where $Q_{k}$ denotes set of frequency samples obtained for an optimum window length.

The same IF estimation strategy was employed in a notable AMC algorithm developed by Lunden [7] and later by the authors of [42]. Similar to [7], we adopted additional median filtering of the IF estimate with a window size of 10 samples. This removes “spikes” which emerge in the estimate for low signal-to-noise ratios (SNRs) [43].

### 4.3. Choi-Williams Distribution Image

Another TFD often used in radar AMC algorithms [7,8,9] is Choi–Williams distribution (CWD), also known as exponential distribution. Its continuous form is given by [44] (p. 92)

(13) $$W_{CW}(t,f)=\int_{-\infty }^{\infty }\int_{-\infty }^{\infty }\frac{\sqrt{\pi \sigma }}{\left|\tau \right|}e^{\frac{-\pi^{2}\sigma {\left(t-u\right)}^{2}}{\tau^{2}}}y\left(u+\frac{\tau }{2}\right)y^{*}\left(u-\frac{\tau }{2}\right)e^{-j2\pi f\tau }\;du\;d\tau ,$$

where τ denotes the lag variable and scaling factor σ controls the tradeoff between cross-terms suppression and frequency resolution. For experiments, we picked a value σ=0.05, likewise to [7]. Here, we determine CWD by multiplying its kernel $g(\nu ,\tau )=\exp(-\nu^{2}\tau^{2}/\sigma )$ with WVD result in the ambiguity domain [44] (p. 245), which may be represented as

(14) $$W_{CW}(t,f)=\int_{-\infty }^{\infty }\int_{-\infty }^{\infty }g(\nu ,\tau )A_{x}(\nu ,\tau )\;e^{-j2\pi (-t\nu +f\tau )}\;d\nu \;d\tau ,$$

(15) $$A_{x}(\nu ,\tau )=\int_{-\infty }^{\infty }\int_{-\infty }^{\infty }W_{WV}(t,f)\;e^{j2\pi (-t\nu +f\tau )}\;dt\;df,$$

where ν denotes Doppler shift, $A_{x}(\nu ,\tau )$ stands for symmetric ambiguity function and $W_{WV}(t,f)$ represents the WVD.

CWD’s inhering ability to attenuate the cross-terms is at the expense of worsened energy concentration. For this reason, in the case of a CWD-based estimator, we do not exploit the time-frequency ridge, but the morphological IF extractor is in its place. Various AMC algorithms [9,39] have used mathematical morphology before. Here, we propose an image processing algorithm based on one described in [8]. The absolute value of the CWD image forms an input image CWD(i,l), where i,l denotes pixel indices. Then, adaptive binarization is performed as follows:

- Calculate the normalized grayscale image with the values from the interval [0,1], as
  $$G(i,l)=\frac{CWD(i,l)-\min CWD(i,l)}{\max\left[CWD(i,l)-\min CWD(i,l)\right]};$$
- Determine the initial threshold as V=[maxG(i,l)+minG(i,l)]/2;
- Set values of all pixels which meet the condition G(i,l)≤V to 0;
- Calculate the Shannon entropy of the resultant image
  $$E\left(G(i,l)\right)=-\sum_{r=1}^{R}P(g_{r})\log_{2}P(g_{r}),$$
  where probability associated with the given pixel value $g_{r}$ is estimated using the 256 bins histogram and R denotes the total number of gray levels;
- Decrease the threshold value by specified step V=V−δV; in this paper δV=0.005 is used;
- Repeat steps c–e until E(G(i,l))≤2V;
- compute final binary image B(i,l), given by
  $$B(i,l)=\{\begin{array}{ll} 1 & G(i,l)\geq V\\ 0 & others \end{array}.$$

After binarization, some isolated noise remains in the image. In order to limit the noise and emphasize the main trend in the IF, the image is additionally treated with morphological opening followed by closing, with a structuring element of increasing size. We employed a square with a size 3⋅3–5⋅5. In the end, the pretreated image may be interpreted again in the time-frequency domain, by simply associating individual pixels with the corresponding time and frequency values B(n,k).

Lastly, an instantaneous frequency estimate is calculated for each time instant as a median of all *K* frequency values corresponding to non-zero pixels

(16) $$\hat{f}(n)=\mathrm{median}\left[B(n,0),B(n,1),\ldots ,B(n,K)\right].$$

**Figure 5:**
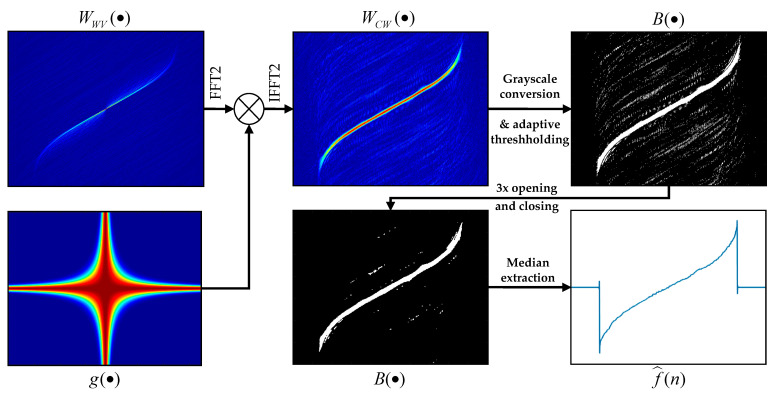
Illustration of the morphological instantaneous frequency (IF) estimator based on the Choi–Williams distribution (CWD) image, for some arbitrary nonlinear-frequency modulated signal (NLFM) immersed in noise with signal-to-noise ratio (SNR) of 0 dB.

The workflow of the CWD-based estimator is depicted in Figure 5. Two subplots on the left-hand side and $W_{CW}(\cdot )$ are related to the transforms described by formulae (14) and (15). The three remaining subplots illustrate the operation of the morphological ridge extractor.

### 4.4. Generalized QML Method

Several authors have remarked that the NLFM waveform may be accurately modeled as a polynomial-phase signal (PPS) [20,24,33]. Thus, in this work we adopt the quasi-maximum likelihood (QML) IF estimator, originally proposed by Djurović and Stanković [45] for PPS signals. However, the initial experiments showed that fast frequency variations on pulse borders prevent signals from being accurately modeled by polynomials only. Consequently, in order to improve the convergence of the model at the borders, we enriched it with tangent component. Finally, a generalized IF model is given by

(17) $$f(t)=\frac{1}{2\pi }\tan\left(\frac{t\pi }{T}\right)\sum_{i=0}^{M}a_{i}t^{i},$$

where $a_{i}$ denotes the respective coefficients of the M-th order polynomial. In experiments, we adopted M=4 in order to avoid overfitting of the model which might arise for higher order polynomials. Furthermore, it is assumed that $a_{1}=a_{3}=0$ to account the fact that frequency modulation law (2) is symmetric.

The concept of this method is to compute a series of IF estimates using discrete short-time Fourier transform (STFT) $W_{SF(h)}(t,f)$, determined with different window lengths from set H. The estimate which maximizes the QML function is considered to be the most accurate. In research, we applied a set of rectangular windows of widths $h_{i}=2^{\left(i+1\right)}$ samples, where i means integer from the interval [1,I], and maximum window length is limited by the number of available signal samples $h_{I}\leq N$.

The algorithm is summarized below, whereby each step is performed for each window length h∈H (for brevity we use continuous time and frequency arguments):

1. Short-time Fourier transform $W_{SF(h)}(t,f)$ is obtained.
2. The initial IF estimate $\bar{f}(t)$ is obtained from the ridge of the spectrogram
  (18) $${\bar{f}}_{h}(t)=\arg\underset{f}{\max}\left|W_{SF(h)}(t,f)\right|.$$
3. Coefficients of the signal model (17) are estimated using least-squares polynomial regression function F(⋅)
  (19) $$F({\hat{a}}_{0},{\hat{a}}_{1},{\hat{a}}_{2})=\underset{a_{i}\in R}{\arg\min}\left({\left|\frac{{\bar{f}}_{h}(t)}{2\pi \tan(t\;\pi /T)}-\sum_{i=0}^{2}{\hat{a}}_{i}t^{2i}\right|}^{2}\right)$$
4. Particular frequency, and then phase is reconstructed on the base of estimated coefficients ${\hat{a}}_{i}$, as
  (20) $${\hat{f}}_{h}(t)=\frac{1}{2\pi }\tan\left(\frac{t\pi }{T}\right)\sum_{i=0}^{2}{\hat{a}}_{i}t^{2i}$$
  (21) $${\hat{\phi }}_{h}(t)=2\pi \int_{0}^{t}{\hat{f}}_{h}(\tau )d\tau$$

Finally, among all calculated estimates ${\hat{f}}_{h}(t)$ we select those that is maximizing the QML function of a form [45]

(22) $$J(h)={\left|\int_{0}^{T}y(t)e^{-j{\hat{\phi }}_{h}(t)}dt\right|}^{2}.$$

Moreover, in simulations, we adopted a slight amendment to the algorithm. It stems from the fact that absolute values of the IF estimate purse to the infinity on waveform edges. Hence, values of ⌈0,2%T fs⌉ boundary samples (where ⌈⋅⌉ denotes ceiling function) on the left and right edge of the pulse are substituted by values of succeeding and proceeding samples adequately.

## 5. Results and Discussion

This section presents the results of conducted experiments. The necessary software was written and executed in MATLAB 2018 and code is available to the reader on request. Algorithms were evaluated using set of complex, baseband test signals buried in noise. The sampling frequency was set to $f_{s}=100\;\mathrm{MHz}$. The number of available signal samples was determined by the signal length. Pulse duration and bandwidths were random variables to reflect the fact that signals intercepted by the intelligence receiver have varying parameters. The distribution of parameters applied in simulation trials is given in Table 2. We consider SNR values from a range typical to ESM systems, i.e., −5 ÷ 30 dB, whereby the SNR is defined as $10\log(P_{x}/\sigma_{\varepsilon }^{2})$ and $P_{x}$ means the average power of a signal estimated using the mean square value. Symbol U(a,b) signifies that the corresponding parameter is evenly distributed within range a to b, Γ(ς,θ) means that it takes a value from gamma distribution with shape ς and scale θ and [a,b] denotes any integer value within given interval.

The accuracy of the algorithms is established using the mean squared error (MSE) evaluated on 250 Monte Carlo trials, i.e.,

(23) $$MSE=\log_{10}\frac{1}{N_{trial}}\sum_{t=1}^{Ntrial}\sum_{n=1}^{N}{\left[{\hat{f}}_{t}(n)-f(n)\right]}^{2},$$

where $N_{trial}$ denotes the number of Monte Carlo runs, ${\hat{f}}_{t}(n)$ is the IF estimate with a length of N samples obtained in t-th trial, and f(n) stands for the true IF law of one of the evaluation signals introduced in Section 2.

### 5.1. General Accuracy

**Figure 6:**
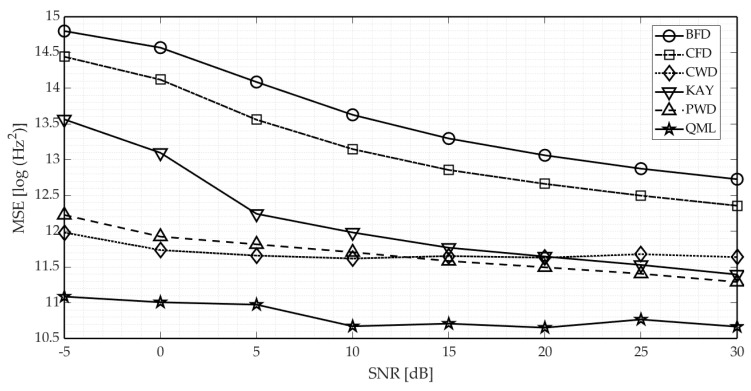
Comparison of estimators’ accuracy in terms of the mean squared error (MSE) for the NLFM A waveform.

In first stage, we compared the accuracy of respective methods, when dealing with LFM as well as various forms of NLFM signals. Figure 6 presents the results obtained for the NLFM A waveform. Our generalized QML algorithm proved to be the most precise among compared methods, across the entire range of SNR, even for its low values (it stems from the fact that STFT is relatively robust to the noise influence despite being biased). The accuracy of both the CWD-based method and data-driven PWD are comparable, albeit the former has a disadvantage in being also reliant on the image processing stage performance. It explains the increase in CWD error, observed for higher SNR values.

For clarity, the results obtained for different waveforms are grouped in Table A1 in Appendix A. However, they follow the same trend.

**Figure 7:**
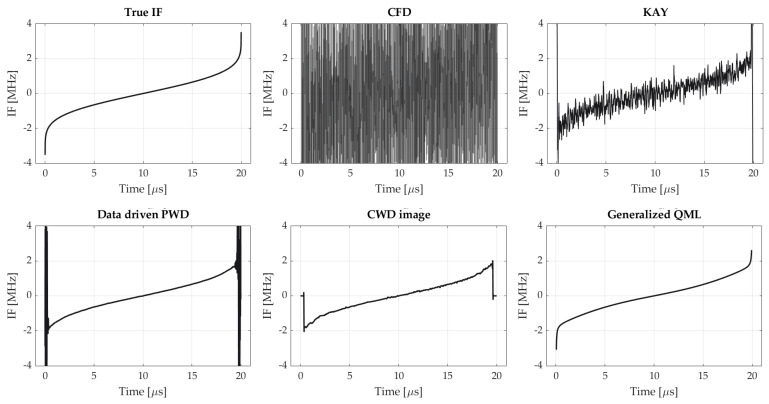
Instantaneous frequency estimates of exemplary NLFM waveform obtained by using different methods compared with true IF-law (backward finite difference method is omitted because similarly to central finite difference it yields noise-like estimate).

Exemplary instantaneous frequency estimates obtained using respective algorithms for SNR = 5 dB are shown in Figure 7.

### 5.2. Accuracy for Different Nonlinearity Levels

**Figure 8:**
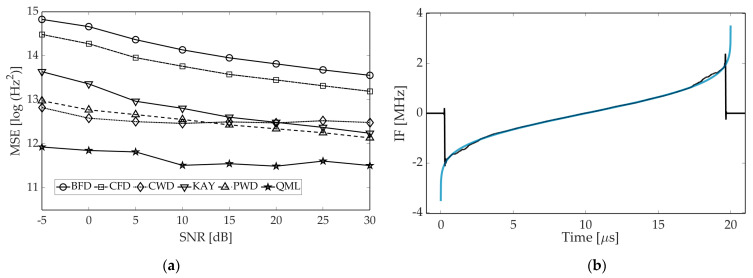
(**a**) The mean squared error achieved by considered IF estimation methods, evaluated on pulse edges only. The error was determined for time samples corresponding to 7% of pulse duration for both pulse ends. The NLFM A waveform was concerned, as on Figure 6; (**b**) illustration of how an inaccurate IF estimate may cause misclassification in the recognition system.

When examining Figure 7, one can discern that for the NLFM signal, the significant part of the error results from the poor accuracy of the estimate on pulse borders. This is reaffirmed in next experiment, whose results are shown in Figure 8a. In this case, MSE was determined entirely on pulse edges. It turns out that the error value is increased by approximately 1 dB, regardless of the applied IF estimation method. In other words, although all estimators perform quite well when the frequency is shifting linearly, their resolution turns out to be insufficient when varying chirp rate come into play (notwithstanding that both PWD and QML employ windows of adaptive size). This is illustrated in Figure 8b. In practice, it may result in the misclassification of the NLFM as an LFM in the AMC algorithm.

**Figure 9:**
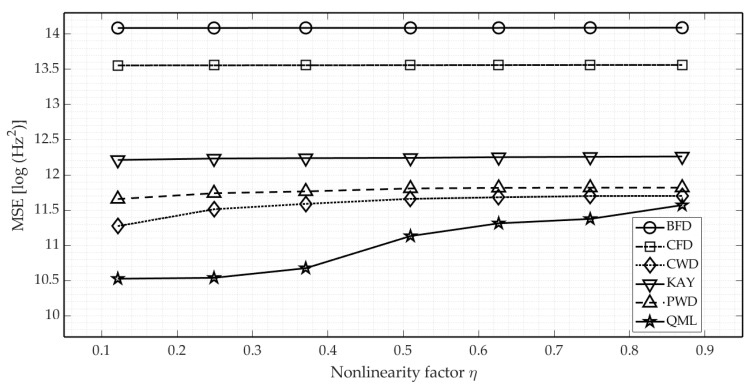
Estimation error in a function of the nonlinearity factor, for a fixed SNR value of 5 dB. In order to adjust the η value, the NLFM B waveform was used with fixed $c_{1}$ and $c_{2}$ coefficients while $c_{3}$ was chosen from range [1.07, 15].

To better understand this effect, we performed additional calculations of the estimation error in a function of the nonlinearity factor. The results are presented in Figure 9.

The noteworthy property of finite difference estimators is their invariance to modulation form, despite being far less accurate when compared with other methods. The same relates to the PWD and CWD estimator. Concededly, both techniques cope slightly better with modulation laws which are close to linear (it emerges that both TFDs exhibit better energy concentration for the LFM), but the decrease in their accuracy for NLFM is minimal.

The proposed parametric QML estimator again revealed itself to be the most precise. The devised frequency modulation model (17) nicely fits the IF-law of NLFM waveforms for nonlinearity factors smaller than 0.4. Above this value, the estimation error rises considerably, but still remains lower in comparison to other methods (it is to be noted that NLFM waveforms of high η will have a strongly diffused spectrum which refrains radar designers from implementing such waveforms in real-life). Still, it is covetable to find another regression model which would better fit the modulation laws of applicable NLFM waveforms with an η value equaling at least 0.6.

### 5.3. Accuracy in Multipath Conditions

**Figure 10:**
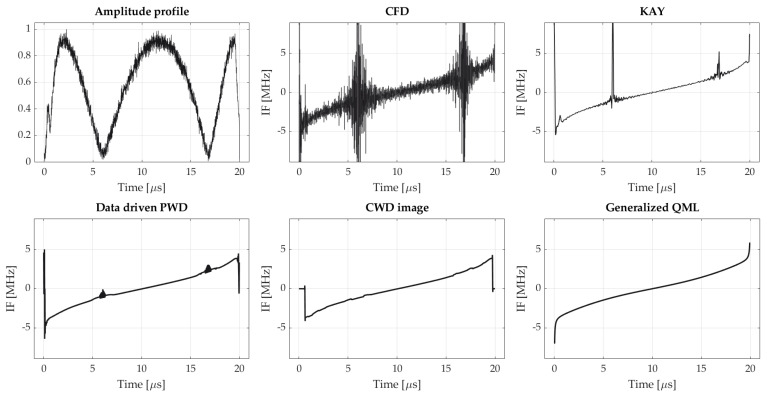
Instantaneous frequency estimates for the NLFM pulse affected by multipath. The envelope of resultant pulse is also shown to give better understanding of the phenomenon. SNR = 20 dB. True IF-law is the same as one presented in Figure 7.

Available reports state that the multipath in ESM receivers appears primarily by quasi-random fluctuations in the pulse amplitude [1]. At the same time, the IF is traditionally considered as a reliable signal feature, which may even carry unintentional modulation on pulse (UMOP) which is characteristic to a certain emitter and thus usable for fingerprinting [46]. Hence, in the next step we surveyed the multipath impact on respective IF estimators. In simulations, we represented the multipath signal as a sum of the discrete-time complex radar signal and its delayed replicas with accordance to formula (5), whereas respective parameters of the model were considered to be random variables with distributions given in Table 2. Complex AWGN noise samples were added to the composite signal, not to respective pulses. Figure 10 illustrates IF estimates obtained using different methods, in the case of a single reflection with an amplitude amounting 0.9 of that of the direct path component is present in the signal. Such a high value of the alternative path component may be assumed as a strong reflection, and may occur for small grazing angles.

**Figure 11:**
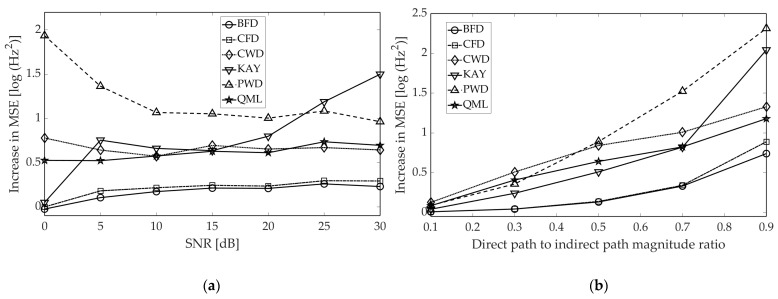
Increase in estimation error in multipath conditions. In order make the results independent of different performance of the estimators at the pulse edges, only the central part of the pulses was taken into account, i.e., 7% of samples on both pulse ends were excluded from the calculation of the MSE. The NLFM A waveform was used. (**a**) Error increase as a function of SNR. Simulation parameters from Table 2 are applied. (**b**) Error increase for a single reflection with fixed ratio of direct pulse to reflected pulse and determined SNR = 15 dB.

It can be noted that for a considered time, the phase and amplitude relationship between the direct-path and reflected pulse, the envelope of the resultant pulse (seen by the ESM system) is severely distorted by destructive interference. For time instants where the amplitude almost completely fades, the estimation error clearly has the biggest value. As it is distinct from other methods, QML does not produce error peaks as it is the only parametric estimator in consideration. To better examine the outcomes of multipath, we performed two simulations aiming at assessing the increase in estimation error for the multipath scenario, compared to a signal free of reflections. The IF of the direct-path pulse was used as the reference for MSE calculation (in fact it is imprecise while true IF is also altered by phase shifts related to the multipath). The results are presented in Figure 11.

Perhaps unsurprisingly, the estimation error grows for all methods, albeit to different extents. Referring to Figure 11a, the error growth is rather unsubstantial for finite phase difference methods, and even declines when the SNR falls below 10 dB. This is due to fact that those estimates are already noise-like, thus MSE approaches its maximum. One can note that KAY is noticeably more vulnerable to the multipath in comparison to BFD and CFD. Although all three estimators rely on the signal envelope, the former “smooths” its values, which results in error amplification when the amplitude exhibits a longer droop.

In the case of TFD-based methods, degradation in accuracy is more evident, which can be noted both from Figure 11a,b. This arises from the cross-terms originating from reflected pulses, as previously remarked in [20]. However, CWD and QML perform slightly better as—when compared to PWD—they have a better ability to suppress the cross-terms, which are further attenuated by the morphological IF extractor and regression procedure, respectively. Nevertheless, dropping performance of the QML is noticeable as the method is designed for slow variations in the instantaneous amplitude. It remains for further study of the improvement of the QML that can be used with high accuracy for multipath signals.

## 6. Conclusions

The choice of proper IF estimator for radar AMC algorithm is application-specific. If a certain ESM system copes with signals with high to moderate SNR values (above 10—15 dB), finite difference-based methods are sufficient. This approach clearly benefits in simplicity—calculations may be performed in a moving window, which facilitates hardware implementation. Among three analyzed estimators of that class, i.e., BFD, CFD and KAY, the latter achieves the lowest variance, which for high SNR approaches that characteristic to TFD-based methods. Still, if the intercept system needs to deal with weaker signals and extra processing power is available, TFD-based algorithms can be the solution, since they are relatively robust to the noise influence.

The highest accuracy can be obtained using parametric estimators. However, they require a priori knowledge about the modulation type present in the signal. This results in three-step signal analysis: firstly, the initial IF estimate may be obtained using general estimators (e.g., CFD), then the AMC algorithm (equivalently human operator) senses the modulation type, and finally the target estimation method suitable for a certain waveform may be employed to refine the initial estimate. Hence, parametric methods are particularly useful when real-time operation is not required, the situation characteristic, e.g., to classical ELINT systems.

The proposed generalized QML method is able adequately reconstruct nonlinear frequency modulation embedded in the waveform. It can be further improved by developing another IF model, e.g., the tansec one described in [47]. There are couple of other prominent estimation methods, such extended generalized chirp transform (EGCT). Still, if one has to deal with a real-time requirement specific to ESM, their applicability is limited due to high computational burden. In this case, a generalized QML algorithm can be used in place.

The provided multipath simulations show that respective IF estimates may be corrupted by reflections. This is accompanied by severe distortions in instantaneous amplitude. What should be regarded in AMC algorithms is that different estimators have different tolerance to this phenomenon. It is an interesting prospect to apply techniques for IF estimation for multicomponent signals [44] (pp. 588–596) in this case, as a pulse affected by the multipath may be considered as a multicomponent signal. All that notwithstanding, we are going make an effort to modify the QML so that it can work more accurately for multipath signals.

Although our simulations concern mainly the NLFM waveform, the presented results may also be referred to different kinds of frequency-modulated radar signals, such as polyphase-coded frequency modulation (PCFM). The introduced notion of the nonlinearity factor may be useful in further research on the synthesis and recognition of NLFM signals.

## Figures and Tables

**Table 1:** Considered radar waveforms.

Waveform	f(t)	Nonlinearity Factor η
LFM ^1^	μt	0
NLFM A [28]	no closed-form	0.48
NLFM B [31] ^2^	$\frac{1}{2\pi }\left\{c_{1}\sinh\left(\frac{t}{c_{2}}\right)+\left(1-c_{1}\right)\left[\frac{2tc_{3}-t^{3}}{{\left(c_{3}-t^{2}\right)}^{\frac{3}{2}}}\right]\right\}$	any, typically ~0.45 when optimized
NLFM C [32]	$\frac{B}{2}\left[\frac{\alpha \tan(2\gamma t/T)}{\tan\gamma }+\frac{2(1-\alpha )t}{T}\right]$	any, 0.35 for α=0.52 and γ=1.47

^1^μ=ΔF/T denotes chirp rate.; ^2^ design parameters $c_{1}\in [0,1]$, $c_{2}\in (0,1)$, $c_{3}\in (1,3)$ are determined during optimization.

**Table 2:** List of simulation parameters.

Experiment	Parameter	Range
all	Pulse duration T [s] Bandwidth B	$\begin{array}{c} U(11\cdot 10^{-6},20\cdot 10^{-6})\\ U(f_{s}/20,\;f_{s}/10) \end{array}$
multipath simulation	Number of reflections Z Reflection coefficient λ Phase shift Δϕ [deg] Detour ΔR [m]	$\begin{array}{c} \left[1,\;10\right]\\ U(0,\;1)\\ U(0,\;360)\\ \Gamma (2.6,\;129) \end{array}$

## Data Availability

We did not report any data.

## Abbreviations

The following abbreviations are used in this manuscript:

AMC	Automatic modulation classification
AMR	Automatic modulation recognition
AWGN	Additive white Gaussian noise
BFD	Backward finite difference
BPSK	Binary phase shift keying
CFD	Central finite difference
CWD	Choi-Williams distribution
EGCT	Extended generalized chirp transform
ELINT	Electronic intelligence
ESM	Electronic support measures
EW	Electronic warfare
GNSS	Global navigation satellite system
IBER	In-bound energy ratio
IF	Instantaneous frequency
LFM	Linear frequency modulation
MIMO	Multiple-input multiple-output
MOP	Modulation on pulse
MSE	Mean squared error
NLFM	Nonlinear frequency modulation
PCFM	Polyphase-coded frequency modulation
PDW	Pulse descriptor word
PPS	Polynomial-phase signal
PWD	Pseudo-Wigner distribution
QML	Quasi-maximum likelihood method
RWR	Radar warning receiver
SPP	Stationary phase principle
STFT	Short-time Fourier transform
TBP	Time-bandwidth product
TFD	Time-frequency distribution
UMOP	Unintentional modulation on pulse
WVD	Wigner-Ville distribution

## Appendix A

**Table A1:** Comparison of IF estimators’ accuracy, for various linear and nonlinear frequency modulated waveforms, in a function of SNR. It is worth noting that the accuracy of finite difference methods is actually independent of the signal form.

Waveform	Method	−5	0	5	10	15	20	25	30
**LFM**	BFD	14.80	14.57	14.09	13.62	13.30	13.06	12.87	12.74
	CFD	14.44	14.12	13.56	13.14	12.86	12.67	12.50	12.38
	KAY	13.58	13.09	12.24	11.95	11.77	11.66	11.55	11.44
	PWD	12.29	11.93	11.79	11.67	11.58	11.49	11.38	11.30
	CWD	12.09	11.82	11.68	11.60	11.65	11.65	11.64	11.66
	QML	11.04	10.77	10.62	10.57	10.46	10.34	10.34	10.35
**NLFM A**	BFD	14.80	14.57	14.09	13.63	13.30	13.06	12.88	12.73
	CFD	14.44	14.12	13.56	13.15	12.86	12.66	12.50	12.36
	KAY	13.56	13.10	12.24	11.98	11.77	11.65	11.53	11.39
	PWD	12.23	11.92	11.82	11.71	11.58	11.49	11.41	11.29
	CWD	11.98	11.74	11.66	11.62	11.65	11.63	11.68	11.64
	QML	11.09	11.01	10.97	10.67	10.71	10.65	10.77	10.66
**NLFM B ^1^**	BFD	14.80	14.57	14.09	13.62	13.30	13.06	12.87	12.72
	CFD	14.44	14.12	13.56	13.14	12.87	12.66	12.49	12.36
	KAY	13.56	13.09	12.25	11.94	11.82	11.67	11.52	11.38
	PWD	12.17	11.91	11.77	11.69	11.58	11.49	11.39	11.28
	CWD	11.81	11.62	11.53	11.55	11.52	11.57	11.53	11.55
	QML	11.26	11.21	10.84	10.77	10.82	10.83	10.82	10.81
**NLFM C**	BFD	14.80	14.56	14.09	13.63	13.29	13.06	12.88	12.73
	CFD	14.44	14.11	13.56	13.15	12.85	12.67	12.51	12.38
	KAY	13.56	13.08	12.25	11.99	11.78	11.65	11.57	11.45
	PWD	12.21	11.90	11.78	11.67	11.56	11.46	11.33	11.29
	CWD	11.95	11.71	11.62	11.59	11.61	11.62	11.61	11.65
	QML	11.04	10.94	10.95	10.76	10.91	10.90	10.95	10.83

^1^ Since optimization procedure assumes optimization, simulation parameters were restricted to discrete values B∈{fs/10,fs/20} and $T\in \left\{11,15,20\right\}\cdot 10^{-6}$.
